# Supporting the parent‐to‐child transfer of self‐management responsibility for chronic kidney disease: A qualitative study

**DOI:** 10.1111/hex.13693

**Published:** 2022-12-23

**Authors:** Ruth Nightingale, Sue Kirk, Veronica Swallow, Gretl A. McHugh

**Affiliations:** ^1^ Language and Cognition Department, UCL Division of Psychology and Language Science University College London London UK; ^2^ Division of Nursing, Midwifery and Social Work, School of Health Sciences, Faculty of Biology, Medicine and Health University of Manchester Manchester UK; ^3^ Department of Nursing and Midwifery Sheffield Hallam University Sheffield UK; ^4^ School of Healthcare, Faculty of Medicine and Health University of Leeds Leeds UK

**Keywords:** adolescent, chronic illness, chronic kidney disease, long‐term condition, parent, qualitative, self‐management

## Abstract

**Introduction:**

As children with long‐term conditions (LTCs) mature, they are usually expected to assume responsibility from their parents for self‐management of their condition. Little is known about what supports families with this handover of responsibility, including the role of healthcare professionals (HCPs). This study aimed to explore what supports young people with chronic kidney disease (CKD) to assume self‐management responsibility and parents to relinquish control.

**Methods:**

A qualitative study, using a grounded theory approach was conducted. Individual and dyadic interviews and focus groups were carried out with 16 young people aged 13–17 years old with CKD, 13 parents, and 20 HCPs. Participants were recruited from two UK children's renal units.

**Findings:**

Building and maintaining trust, fostering positivity, learning from mistakes, forming partnerships and individualized support, facilitated the transfer of self‐management responsibility. However, HCPs' focus on developing partnerships with young people meant some parents felt excluded, highlighting uncertainty around whether support should be child‐ or family‐centred. Although tailored support was identified as critical, aspects of local service provision appeared to impact on HCPs' capacity to implement individualized approaches.

**Conclusion:**

This study has identified what supports the handover of responsibility, and, importantly, HCPs' current, and potential role in helping young people to assume responsibility for managing their LTC. Further research is needed to explore how HCPs' involvement balances child‐ and family‐centred care, and how HCPs can adopt personalized, strengths‐based approaches to help ensure the support that families receive is tailored to their individual needs.

**Patient or Public Contribution:**

Patient and public involvement was integrated throughout the study, with young adults with CKD and parents who had a child with CKD actively involved in the study's design and delivery.

## 1 INTRODUCTION

Self‐management has become an increasingly important aspect of health care across all age groups, due to the growing prevalence of long‐term conditions (LTCs).^1^ Although definitions of self‐management vary, Lorig and Holman^2^ suggest it involves medical, role and emotional management to enable the individual ‘to manage the symptoms, treatment, physical and psychosocial consequences and lifestyle changes inherent in living with a chronic condition’ (p. 178). As children are usually dependent on, or share condition management with their parents, alternative terms such as ‘supported self‐management' and ‘responsibility sharing’ have been used in childhood LTCs,^3^, ^4^ As children mature, they are expected to assume responsibility from their parents for self‐management of their LTC.^5^ However, this expectation has been challenged, and studies suggest that for some families, shared parent‐child management is preferable to the young person managing their LTC independently.^6^, ^7^

Healthcare policy and research focuses on adolescence and the transition between child and adult services as the main developmental phase to acquiring self‐management skills.^8^, ^9^ Consequently, healthcare professionals (HCPs) tend to view the assumption of self‐management responsibility as a process that starts when the young person is around 13 years old and ends with the transfer to adult services.^10^ Studies suggest, however, that families can start this process at an earlier developmental stage,^6^, ^11^, ^12^ and some guidelines recommend that HCP support to develop self‐management skills should start in early childhood.^4^ This uncertainty around the optimal time for children to assume responsibility is compounded by studies highlighting adolescents' difficulties engaging in self‐management, resulting in adverse consequences for their health.^13^ Additionally, the conflation between children's age and competency and the tendency of HCPs to view children as a homogenous group,^14^, ^15^ underlines the need for individualized support with the transfer of responsibility.

An integrative review that explored the parent‐to‐child transfer of self‐management responsibility found that this transfer was a complex, individualized process.^16^ The review identified how children and parents adopted various strategies to facilitate the transfer of responsibility, but there was limited evidence about the approaches used by HCPs and ambivalence around what was helpful. Where research explored what supported children to assume responsibility, this was primarily from the perspectives of children and parents; the views of HCPs were noticeably absent. Due to this gap in the literature, the review suggested further research was needed with all key stakeholders, including children, parents and HCPs, to gain a better understanding of the transfer process and what supports families with the handover of responsibility.

Research around the transfer of self‐management responsibility has mostly focused on diabetes and asthma, two of the most prevalent childhood LTCs.^16^, ^17^ As LTCs differ in severity and self‐management demands can vary, a condition‐specific approach can be useful when studying the parent‐to‐child transfer of responsibility.^18^ Therefore, this study focused on chronic kidney disease (CKD), a complex LTC related to irreversible kidney damage, with a wide range of causes and complications.^19^ Children with CKD can be classified by stages 1–5, based on the rate at which the kidneys filter waste products; stage 5 indicates end‐stage kidney disease, which means renal replacement therapies, such as dialysis or kidney transplantation, are needed.^20^ Although CKD shares some self‐management tasks with other LTCs, condition‐specific demands include renal diets, fluid restrictions or targets and dialysis, either carried out in a hospital or home setting. In the United Kingdom, 13 specialist renal centres manage the care of children with CKD stages 3–5.^21^ As the majority of CKD management tasks are undertaken outside of the renal centre (e.g., in the child's home or school), and because CKD is a lifelong condition, child and family assumption of management responsibility is critical.

Studies suggest children, especially during adolescence, experience difficulties engaging in CKD self‐management.^22^ Adolescents have higher levels of kidney transplant loss compared to younger children and adults^23^ and less than 20% of adolescents on dialysis were perceived by HCPs to have assumed self‐management responsibility at transfer to adult care.^24^ While the literature suggests the parent‐to‐child transfer of self‐management responsibility is an important aspect of children's development, there is limited research on this transfer process involving children with CKD, and, crucially, how the process can be supported. Therefore, this study aimed to address this gap by exploring what supports young people with CKD to assume self‐management responsibility and parents to relinquish control.

## 2 METHOD

The study used a constructivist grounded theory methodology.^25^ Grounded theory is useful in exploratory research, as it aims to construct a theory that offers in‐depth understanding and explains the phenomenon being studied.^26^

### 2.1 Sampling and recruitment

Participants were recruited from two UK children's kidney units. Purposive sampling was initially used as the aim was to achieve maximum variation in relation to (1) young people aged 13–18 years old with CKD stages 3–5 and their parents/carers, and (2) HCPs from a range of disciplines in the renal multidisciplinary team. As the study progressed, theoretical sampling was used to sample young people with CKD stages 3–4, to generate data to elaborate and refine the emerging categories. One clinician from each of the kidney units identified potential participants and gained consent for R. N. to provide them with study information. A total of 49 participants took part in the study comprising 16 young people (Table 1), 13 parent/carers (11 mothers, 1 step‐father, 1 carer) and 20 HCPs (5 renal paediatricians, 4 nurses, 4 social workers, 3 clinical psychologists, 3 play workers, 1 dietitian).

**Table 1:** Characteristics of young people (*n* = 16)

Young people's characteristics	Girls (*n* = 9)	Boys (*n* = 7)	Total
Age			
13	1	2	3
14	1	3	4
15	2	1	3
16	4	1	5
17	1	0	1
Ethnicity			
White	4	3	7
South Asian	3	2	5
Black	2	1	3
Other	0	1	1
CKD stage/treatment			
Pre‐emptive transplant	0	3	3
Dialysis	4	3	7
In‐centre haemodialysis	1	3	4
Home dialysis	3	0	3
Transplant	5	1	6

Abbreviation: CKD, chronic kidney disease.

### 2.2 Data collection

Semi‐structured interviews and focus groups were conducted to generate data. Young people and parents were offered the opportunity to be interviewed together or separately, and HCPs participated in either individual interviews or focus groups (Table 2).

**Table 2:** Data collection methods

Method	Type of participant/number	Length (range, in minutes)
Individual interview (*n* = 21)	Young people = 7 Parents = 4 HCPs = 10	24–78
Paired interview (*n* = 9)	Young people/parent dyads = 9	46–93
Focus group (*n* = 2)	13 HCPs	
	Focus group A = 9 × HCPs (renal paediatricians = 3; clinical psychologists = 2; social workers = 2; nurse = 1; play worker = 1). 3 of these HCPs also took part in an individual interview	46
	Focus group B = 4 × HCPs (social workers = 2; clinical psychologist = 1; play worker = 1)	54

Abbreviation: HCP, healthcare professional.

R. N. conducted all data collection, although the larger focus group (A) was co‐facilitated by V. S. Interviews and focus groups took place in person in the family home or hospital setting, or by telephone and were guided by a topic guide. As part of theoretical sampling, topic guides were revised as the study progressed (Supporting Information: 1). Interviews and focus groups were digitally recorded and transcribed verbatim. To address some of the methodological and ethical issues of conducting research with children, task‐based methods were used to generate data.^27^ For example, in later interviews, participants were asked to consider the suggestions generated during earlier interviews around what supported the transfer of responsibility. Each individual suggestion was written on a piece of card, which was handed to participants, with the request that they consider each suggestion in turn.

### 2.3 Data analysis

Data collection and analysis were conducted concurrently, using an iterative, inductive process. Initial codes were developed by line‐by‐line coding, with the aim of identifying actions and processes in the data. Focused coding, in conjunction with constant comparison, involved evaluating the initial codes to identify analytical, and theoretical categories.^25^ A supplementary approach was used to analyse how interaction contributed to data generation in the paired interviews and focus groups.^28^ NVivo11 was used to code and manage data. To ensure trustworthiness and credibility, reflexivity and regular discussion between authors were incorporated into the analytic process.

### 2.4 Patient and public involvement (PPI)

PPI was integrated throughout the study, with two young adults with CKD and two parents of young people with CKD involved in the study's design and delivery. Table 3 summarizes the PPI at different stages of the study.

**Table 3:** PPI contributions

Stage of study	Advice sought	Methods
Initial research idea/before study started	Relevance of research idea; study methods; plain English summary for funding application	Online meeting
		Email
Applying for ethical approval	Participant information leaflets	Email
Data collection	Topic guides and task‐based methods used during interviews	Face‐to‐face meeting
		Email
Data analysis	Discussion of study findings	Online meeting
Dissemination	Plain English summary of study findings for participants	Email

Abbreviation: PPI, Patient and public involvement.

The impact of PPI on the study was manifold. For example, during discussions, none of the PPI contributors used the term ‘self‐management’, instead describing young people ‘being in control’ and ‘taking charge’ of their health care; this had a significant impact on the language used with participants throughout the study, especially during data collection. PPI contributors' advice to change some of the language and design of the participant information leaflets made the leaflets more accessible and, through provision of improved information, potentially supported participants to make an informed decision about whether to participate.^29^ Topic guides were revised based on feedback to: ask additional questions to explore other aspects of self‐management PPI contributors thought relevant; alter existing questions so they were easier to understand; and adjust the order of the questions. Consultation with PPI contributors about study findings suggested the emergent categories and theory resonated with their own experiences of the transfer of CKD self‐management responsibility.

### 2.5 Ethical issues

Participants were provided with age/developmentally appropriate information, and all provided informed assent/consent. Participants were assured of confidentiality and anonymity. In the data extracts presented, participants are identified by the type of participant (young person, parent, HCP) and the participants' numerical study identifier (1–20). The young person's age and gender are included in the data extracts to provide contextual information.

## 3 RESULTS

**Figure 1:**
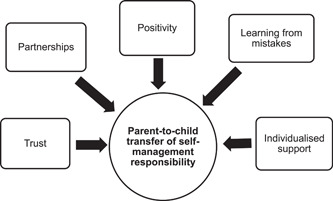
Supporting the parent‐to‐child transfer of self‐management responsibility

A grounded theory, *shifting responsibilities*, was constructed from the data, consisting of a core category (*shifting responsibilities*) and two connected subcategories (*developing independence* and *making changes*). Further details about the grounded theory, core category and subcategories have been reported previously.^12^ This paper focuses on a specific aspect of the second subcategory, making changes, to explain how young people's, parents' and HCPs' adjustments to their behaviour and communication supported the parent‐to‐child transfer of self‐management responsibility. This included behaviour and communication that: built and maintained trust; formed partnerships; fostered positivity; supported learning from mistakes, and was responsive to young people's and parents' individual preferences and needs (Figure 1). A gradual transfer, developing a routine, and connecting with others with CKD were also perceived to support the transfer process and have been described elsewhere.^12^

### 3.1 Building and maintaining trust

Young people, parents and HCPs perceived trust was needed for the transfer of self‐management responsibility. This included trusting relationships between young people and their parents and between the young person–parent dyad and HCPs. Additionally, some young people suggested trusting themselves was an aspect of assuming responsibility. When young people were able to demonstrate they could consistently engage in self‐management, parents started to trust that their child could be relied on to perform self‐management:

It became a habit, I got good at taking them [medication], there was that trust. Then I stopped taking them. I think that trust is there again, but when I stopped taking them, I was obviously not being responsible. (YP1, 14‐year‐old girl)

Parents adjusted their behaviour and communication as trust was built with their child; for example, they reduced how often they reminded their child to take their medication or monitored their renal diet. Trust was essential for parents to feel able to relinquish control. However, as the quotation above highlights, maintaining trust was difficult, especially if the young person disengaged, even temporarily, from self‐management. When trust was lost, the transfer process was disrupted as parents tended to reassume responsibility and an increased level of control.

Some HCPs' perceived they had a role in supporting parents to start trusting their child was able to self‐manage. This included identifying opportunities where the young person would be able to demonstrate to their parents that they could be trusted, such as connecting themselves safely to their dialysis machine, or following their renal diet when outside the family home:

With the diet, one thing happens at home, and another thing happens at school or when they're out with their friends. One way that I tackle it, is for them to take more responsibility for what happens when they're not at home first. If they can show their parents that they're managing well when they're out on their own, and the parents can trust them to make the right decisions, then that shows them that they are capable of managing … it's trying to build up the trust between the child and their parents. (HCP2)

Trusting relationships between the young person–parent dyad and HCPs were perceived as supporting the transfer of responsibility:

If they trust in you, I think that's very helpful. I've looked after most of these people for the last 14 years, I'm a familiar face. We've got a relationship, we've built up trust over time, that really helps. To analyse the problems, the young person has got to be open first. (HCP8)

This quotation suggests that trust needed to be two‐way, that HCPs needed to be able to trust families, as well as young people and parents trust HCPs. There was a sense that as young people assumed self‐management responsibility, they needed to be ‘open’ with HCPs, which was more likely if there was a trusting relationship. Some young people described how being able to trust themselves, or having confidence in their ability to manage their condition, was part of assuming responsibility. Their accounts suggested that this impacted how much their parents were able to trust them and relinquish control:

I can't trust myself with food, because I like a lot of food that I'm not supposed to eat. Sometimes I won't be able to contain myself from not eating it. My mum, she cares too much about me to stop reminding me about the things I eat, so she won't hand me that responsibility that easily. (YP15, 15‐year‐old boy)

Approaches used to support young people to trust themselves and develop self‐confidence, included HCPs and parents acknowledging when young people were managing their condition. This will be discussed in more detail in Section 3.3, fostering positivity.

### 3.2 Forming partnerships

Partnerships between the young person, their parents and HCPs were perceived to support the transfer of self‐management. Young people and parents described how ‘teamwork’, which included undertaking self‐management activities together, supported young people to become increasingly involved in managing their CKD. HCPs adopted a range of approaches to encourage partnership including directing communication primarily at young people rather than parents; exploring young people's concerns and their motivation to assume responsibility; joint goal‐setting; findings solutions together; acting as an advocate for the young person and helping young people to negotiate with their parents around the transfer of responsibility. Young people appeared to value being treated as an equal; they described how interactions with HCPs that encouraged partnership, supported their assumption of responsibility:

It's a two‐way thing. They [HCPs] want your take on it, because they don't want to be saying things and then me leave and be, ‘Forget that. I'm not doing that’. They ask our opinions, how it would work. They are very supportive in that way. It's your opinions and their opinions, but they mostly want your take on it, so you can help them understand. I like the independence, they're treating me like an adult rather than a kid. (YP1, 14‐year‐old girl)

Although most HCPs encouraged young people to attend appointments on their own, there were conflicting views among HCPs around how much parents should be included and whether they were a barrier or facilitator to young people taking responsibility for condition management. The few young people who had attended appointments on their own appreciated having the opportunity to focus on issues important to them and talk more openly with HCPs, compared to when their parents were present. Parents, however, appeared more ambivalent about HCPs' decisions to include or exclude them from consultations; while they seemed to accept that HCPs forming a partnership with their child was a necessary stage in their child assuming responsibility, they also struggled with relinquishing control. Some HCPs emphasized the need to partner with the young person–parent dyad and perceived parents' involvement was critical to supporting young people to assume responsibility:

It does need to be in tandem because they are closely entwined. The danger of doing it in isolation is that the young person comes home and goes, ‘Mum I've talked to this nurse, I want to take my own meds’, and the parent goes, ‘No bloody way!’ Unless you're doing it together, I mean it could work, but it's going to be more successful if you're doing it as a combined approach. (HCP17)

### 3.3 Fostering positivity

Young people, parents and HCPs described how the transfer of responsibility was often a difficult process, in particular when young people struggled to integrate self‐management into their daily life. Therefore, behaviour and interactions that fostered positivity, such as acknowledging when the young person had been able to manage their condition, and focusing on what was going well, were perceived to support the transfer of self‐management. Parents, in particular, emphasized the importance of keeping positive, even when their child was struggling with self‐management:

Sometimes she'll [child] say, ‘I'm doing well with my tablets, aren't I?’ I'll be like, ‘Oh, yes’. I try to be positive about it, but I can't say if she's had any tablets yesterday. I try to look at the positive stuff, she could be a lot worse than what she is, behaviour wise, but it is a concern to me. (Parent7, 16‐year‐old girl)

HCPs' accounts suggested they were aware of the need to acknowledge a young person's strengths. However, there was a sense this rarely happened, as appointments tended to focus on problems, including the young person's difficulties with assuming responsibility:

Sometimes patients do nine tasks out of ten really well, but the focus in clinic will be on the one they're not doing, which is disheartening on the young person, because they probably really tried, and it's the one thing that they've not managed to stay on top of. Conversations tend to be so negative, that it puts them right off trying again. Somebody needs to say, ‘Well done for doing your medicine, turning up today, engaging in your healthcare, but we need to work a little bit on…’. (HCP1)

As this extract suggests, HCPs making changes to their interactions with young people to acknowledge what they had achieved and provide positive feedback was perceived to support young people's motivation to continue engaging in self‐management.

### 3.4 Supporting learning from mistakes

When young people had difficulties with assuming self‐management responsibility, learning from mistakes was perceived to be helpful. Some young people acknowledged the impact on their health when they stopped engaging in self‐management, and this prompted them to resume responsibility:

I definitely learnt from my mistake. I keep my water bottle near now. I make sure I'm keeping on top of things. I have all my medications properly, and check and double‐check that I've got all my medications. (YP14, 16‐year‐old girl)

Although parents were aware of the potential risks of their child making mistakes with self‐management, they accepted making mistakes was ‘normal’ and could provide opportunities for their child to learn:

I'd tell parents with teenage children, when they make mistakes, let them see. Let them understand that sometimes they will make mistakes. Don't teach them there's no mistake, no, then you make them so rigid, let them be free with you. Tell them it's a mistake and this is the repercussion, so they know. (Parent3, 15‐year‐old girl)

As this extract suggests, acknowledging that young people would make self‐management mistakes could potentially encourage young people to be ‘free’ or honest with their parents when they were struggling with self‐management. HCPs accounts also indicated how learning from mistakes could facilitate the transfer of responsibility. Some HCPs described discussing with families how mistakes could provide opportunities for young people to develop an understanding of the consequences of their self‐management decisions:

Being a teenager is about making mistakes, it's learning from your mistakes. But we don't want them to make mistakes that cause them harm … I talk to the family, I say making mistakes is the learning process, let them make mistakes safely, not letting them make any mistakes is not safe. (HCP8)

However, as this quotation highlights, the emphasis was on making mistakes ‘safely’ due to the awareness that some self‐management mistakes could have a significant impact on the young person's health.

### 3.5 Individualizing support

Young people, parents' and HCPs' accounts suggested that the transfer of responsibility was completely individualized to each family. Contextual issues, such as the: young person's chronological and developmental age; family relationships and physical and social environment, interacted with and influenced the transfer process. A young person's progression through the CKD stages and the condition‐specific self‐management requirements, such as starting dialysis or receiving a kidney transplant, were also perceived to impact the young person's assumption of responsibility and parents' willingness to relinquish control. During a dyadic interview, a 16‐year‐old girl and her mother discussed how responsibility shifted after she had received a transplanted kidney:

*Young person*: Before my transplant I was responsible for taking my tablets of an evening, and you would just know. You wouldn't even—,

*Parent*: She only took two tablets. She took them at night and at that point I never used to check in. Now and again I used to say, ‘Have you taken your tablets?’ when I said goodnight, but it's not like it is now. I think it's the importance of the tablets, because tacrolimus [immunosuppressive medication], if you forget it, it's massive … I was a lot more slapdash then. (YP8, Parent 8, 16‐year‐old girl)

HCPs accounts indicated they were aware that the transfer of responsibility was experienced differently by each family. The importance of individualizing support to each family's needs was discussed in focus groups, as HCPs generated ideas around what facilitated the transfer of self‐management:

*HCP8*: For some people, meeting other patients would be hell, for some it would be great … there isn't one size that fits all.

*HCP1*: It's tailoring it. Like you say, some people wouldn't engage, some don't like digital technology, but they'd like the face‐to‐face. It's finding what fits.

Although HCPs believed support needed to be individualized, national and local transition guidance around young people moving from paediatric to adult services, underpinned service provision. Consequently, HCP involvement in the transfer of responsibility tended to start when young people were around 13 years old and finished when they transferred to adult services. Some HCPs accounts revealed their frustration that the young person's chronological age, rather than their ability to self‐manage, determined when they moved to adult services:

*HCP8*: We are driven by age … that drives when we do transition rather than the patient.

*HCP11*: It depends as well where you work. We have a [NHS] Trust that mandates that we move patients over at the age of 16 … but there are other Trusts where between 16 and 19, young people are offered a choice, ‘Do you want to go to paediatric services, or move up to adult services?’ So how we practise as clinicians is dictated by the management who decide how they want to do things within this Trust. (A National Health Service (NHS) Trust is an organizational unit in England and Wales that provides health services, and generally serves either a geographical area or a specialized function).

These extracts highlight potential tensions between HCPs' belief in the need for individualized support and what they were able to provide in practice.

## 4 DISCUSSION

Previous studies have explored the parent‐to‐child transfer of self‐management responsibility, but little is known about what support young people and parents' need as responsibilities shift.^16^ This study contributes to knowledge by identifying what facilitates this transfer process, and, importantly, HCPs' current and potential role in helping both young people to assume responsibility, and parents relinquish control. Findings suggest there were similar views among young people, parents and HCPs about what supported the transfer of responsibility. However, some tensions appeared to be evident, in particular around the formation of partnerships between HCPs and young people that excluded parents, and the provision of individualized support. By highlighting what facilitates the transfer of responsibility, study findings both support and extend the existing literature, and have implications for practice.

Behaviour and communication that built and maintained trust were perceived to help the transfer process. This finding supports existing research that found parents needed to trust their child to relinquish control.^30^, ^31^ However, by exploring HCPs' perspectives, this study extends the current understanding of the HCP role, suggesting HCPs could contribute to the development of trusting parent‐child relationships. Previous studies recognized that situations, when the child was away from the family home (e.g., to attend school, or socialize with friends), could be anxiety‐provoking for parents, and therefore, recommendations were made that HCPs should provide reassurance to parents about their child's self‐management ability.^32^ In contrast, this study's findings suggest that by actively identifying situations when the young person has the opportunity to demonstrate to their parents they can be trusted to engage in self‐management, HCPs can help build and maintain trust. The importance of trusting relationships between young person–parent dyads and HCPs has been highlighted in previous research. Sullivan‐Bolyai et al.^33^ found parents lost trust in HCPs when HCPs believed the deterioration in young people's health was a consequence of parents' transferring responsibility to the child before they were ready. The inclusion of HCPs in this study, however, extends knowledge in this area by suggesting trust is two‐way, as young people–parent dyads need to trust HCPs, and HCPs need to trust families.

Some young people in this study believed they needed to be able to trust themselves to assume self‐management responsibility. This suggestion that young people with CKD benefit from developing confidence and belief in their own ability aligns with the concept of self‐efficacy.^34^ Although the literature proposes that enhancing self‐efficacy can facilitate young people assuming responsibility,^18^, ^35^, ^36^ there is limited empirical research to support this. Colver et al.^9^ suggest HCPs should encourage self‐efficacy and recommend further research ‘to identify the most effective and efficient ways to promote young people's knowledge and confidence in the management of their LTC’ (p. 77). By identifying approaches that can support young people's belief in their self‐management ability, such as fostering positivity and connecting with others with CKD,^12^ this study's findings have implications for practice.

Young people, parents' and HCPs' perceived partnerships supported the transfer of responsibility. Previous research has highlighted the importance of collaborative child–parent relationships, as young people are more likely to learn self‐management from their parents, rather than HCPs.^22^, ^37^ Participants' accounts suggest that HCPs formed partnerships primarily with young people, rather than the young person–parent dyad, as they perceived this encouraged young people to assume responsibility. As reported previously, HCPs tended to view the transfer of responsibility as part of the transition between child and adult services.^12^ Consequently, UK transition guidance shaped HCP involvement, including the importance of young people attending clinic appointments without their parents.^8^, ^38^ Consistent with previous research, young people in this study valued meeting with HCPs on their own, as they felt more able to talk openly without their parents present.^22^ While some parents were positive about their children attending appointments without them, others struggled with being excluded and wanted to be kept informed.^39^ The conflicting views among HCPs about whether parents are a facilitator or barrier to the transfer process and parents' ambivalence about their inclusion or exclusion from consultations,^40^ extend the debate around whether HCP involvement should be child‐ or family‐centred.^41^ Although it has been recommended that triadic collaboration is fostered between young people, parents and HCPs during the transfer of responsibility,^9^, ^42^ only a few HCPs in this study seemed to view parents as supporting the assumption of responsibility. Therefore, few aimed to form a partnership with the young person–parent dyad. The uncertainty around how HCPs balance child‐ and family‐centred care during the parent‐to‐child transfer of responsibility indicates further research is needed.

Parents and HCPs perceived the transfer of responsibility was supported by fostering positivity. Only a few previous studies exploring diabetes self‐management identified positive reinforcement and offering rewards as helping young people to assume responsibility.^33^, ^43^ However, as neither of these studies included HCP participants, further research exploring how HCPs can adopt a ‘strengths‐based approach’, as recommended by UK transition guidance, is needed.^38^ Consistent with previous studies, young people learnt from making mistakes with self‐management.^44^, ^45^ Parents and HCPs were aware, however, that some mistakes could have a significant impact on the young person's health and, as a result, there was ambivalence about learning through trial and error.^46^ Although the existing literature recommends HCPs increase opportunities for experiential learning so young people can learn from the mistakes they make,^33^ there is limited evidence to suggest that HCPs have utilized this strategy. Potentially due to including HCP participants, this current study extends knowledge in this area, finding that HCPs discussed with parents how making mistakes ‘safely’ was part of their child assuming responsibility.

In this study, HCPs described the importance of tailored support to meet the individual needs of young people and parents. Previous literature has discussed the need for HCPs to consider children as individuals and avoid having a uniform policy around when, and how the transfer of responsibility occurs.^15^, ^47^ However, as a consequence of UK transition guidance underpinning HCPs support to young people assuming responsibility, HCP involvement tended to be service‐led, rather than based on family needs.^12^ This highlights a potential tension between HCPs' beliefs that support needs to be individualized and what occurs in practice. Although guidance recommends HCPs adopt individualized or personalized approaches,^1^, ^4^ there is limited evidence around how HCPs use these approaches in practice to support the transfer of responsibility. Further research to explore how HCPs construct and implement individualized support to facilitate the parent‐to‐child transfer of responsibility is needed.

### 4.1 Strengths and limitations

Having PPI to advise on the design and conduct of this study was a major strength and impacted on the quality and relevance of its findings. An equal focus on HCPs' perspectives, alongside those of young people and parents, assisted with gaining an in‐depth and holistic understanding of what supports young people to assume self‐management responsibility. Although there was diversity in the sample, especially in relation to young people's age, ethnicity and CKD stage/treatment and HCPs' discipline, selection bias may have occurred due to reliance on clinicians in the kidney units for recruitment. Diversity could have been increased further through the recruitment of a greater number of fathers. Dyadic and focus groups can generate unique ethical and practical challenges, as power relations and family/group dynamics can potentially inhibit some participants from speaking.^48^, ^49^ However, adopting techniques such as task‐based methods, the researcher aimed to encourage young people and ‘quieter’ group members to contribute to discussions.

## 5 CONCLUSION

This study has explored what supports the parent‐to‐child transfer of self‐management responsibility for CKD. Study findings have contributed to knowledge, and, importantly, have identified HCPs' current and potential role in facilitating young people to assume responsibility and parents to relinquish control. These new insights have implications for practice, highlighting how families would benefit from individualized support that helps to: build and maintain trust, form partnerships that include parents, foster positivity and support learning from mistakes. Conflicting views around whether parents are a barrier or a facilitator to young people assuming responsibility indicate further research is needed to understand how HCPs can balance child‐ and family‐centred care when supporting the transfer process. Finally, further research to explore how HCPs can adopt personalized and strengths‐based approaches in practice would help ensure the support that families receive is tailored to their individual needs.

## CONFLICT OF INTEREST

The authors declare no conflict of interest.

## ETHICS STATEMENT

Approval was obtained from the UK Health Research Authority (226365), a National Health Service (NHS) Research Ethics Committee (18/YH/0210) and the NHS Trust Research and Development Departments.

## Supporting information

Supporting information.

## Data Availability

Research data are not shared.
