# Supplementary material for: Supporting the parent‐to‐child transfer of self‐management responsibility for chronic kidney disease: A qualitative study
Source: Health Expect. 2022 Dec 23;26(2):683–92. doi: 10.1111/hex.13693 (PMC10010075; doi:10.1111/hex.13693)
Supplement: Supplementary file 1 — Supporting information. [file HEX-26--s001.docx]

**Supporting Information 1: Initial topic guide (young people)**

1. Could you tell me about a typical school/college day – what kind of things are you doing, starting when you get up?

- How does having a kidney condition affect your day?
- How are things different at the weekend?

1. What do you do to take care of your kidney condition? e.g. medication, food, drink, sleep, exercise.

- What other things do you do to keep healthy?

1. How do you find it taking care of your kidney condition?

- What is easy/difficult?
- What else would help you take good care of yourself?

1. How do your parents help you to take care of your health?

- What are they doing?
- Who else helps you? e.g. other parent, other family members, friends, school, HCPs.
- What works/doesn’t work about sharing responsibility with other people?

1. With looking after your health, how has it changed over time?

- When did you start to take over care for your kidney condition?
- How did you begin to take over care?
- What prompted you to start taking over care?

1. As you have become more in control of looking after your health, what/who has helped you?

- What wasn’t been helpful?

1. What has your experience been like with health professionals from the kidney team?

- Were they involved in helping you become more in charge?
- How?
- How could things be improved?
- Have you attended a transition clinic? How have you found this? How is it different to the clinic you went to when you were younger?

1. As you have started to take control of your health, are there other areas in your life where you have become more independent? e.g. at home, school/college
2. How do you feel about taking/being in control of your health?
3. With looking after your health, how will things change as you get older?

1. Is there something else you think I should know about how teenagers take over care for their kidney condition?

**Revised topic guide (young people)**

1. Could you tell me about a typical school/college day – what kind of things are you doing, starting when you get up?

- How does having a kidney condition affect your day?
- How are things different at the weekend?

1. With looking after your health, how has it changed over time?

- When did you start to take over care for your kidney condition?
- How did you begin to take over care?
- What prompted you to start taking over care?

1. As you have become more in control of looking after your health, what has helped you?

- Who has helped you? e.g. family, health professionals, friends, teachers
- How did they help you?
- What other things might have been helpful?
- What hasn’t been helpful?

1. Would you like more help with learning to take over care for your condition?

- If yes, can you tell me about what would be helpful? *[Use prompt cards, ideas from earlier interviews with young people, what do they think?]*
- What kind of help?
- Who could provide this help?
- When? e.g. at certain times – change in condition/treatment; changing schools
- Where? e.g. hospital, home, school etc
- If no, can you tell me your reason for not wanting more help?

1. How do you think your parents felt as you became more involved in your care?
2. Do you think your parents would like help with letting go of doing the care?

- If yes, can you tell me about what would be helpful?
- What kind of help?
- Who could provide this help?
- When?
- Where?
- If no, can you tell me your reason for not wanting more help?

1. What advice would you give to another teenager with a kidney condition who was going to become more in control of looking after their health?
2. Is there anything else I should know about how teenagers and parents could be helped with handing over care for their kidney condition?
